# Interplay of Oxidative Stress, Gut Microbiota, and Nicotine in Metabolic-Associated Steatotic Liver Disease (MASLD)

**DOI:** 10.3390/antiox13121532

**Published:** 2024-12-14

**Authors:** Irene Mignini, Linda Galasso, Giulia Piccirilli, Valentin Calvez, Fabrizio Termite, Giorgio Esposto, Raffaele Borriello, Luca Miele, Maria Elena Ainora, Antonio Gasbarrini, Maria Assunta Zocco

**Affiliations:** CEMAD Digestive Diseases Center, Fondazione Policlinico Universitario “A. Gemelli” IRCCS, Università Cattolica del Sacro Cuore, Largo A. Gemelli 8, 00168 Rome, Italy; irene.mignini@guest.policlinicogemelli.it (I.M.); linda.galasso@guest.policlinicogemelli.it (L.G.); giulia.piccirilli01@icatt.it (G.P.); valentin.calvez@guest.policlinicogemelli.it (V.C.); fabrizio.termite01@icatt.it (F.T.); giorgio.esposto@guest.policlinicogemelli.it (G.E.); raffaele.borriello@unicatt.it (R.B.); luca.miele@policlinicogemelli.it (L.M.); mariaelena.ainora@policlinicogemelli.it (M.E.A.); antonio.gasbarrini@unicatt.it (A.G.)

**Keywords:** metabolic-associated steatotic liver, nicotine, cigarette smoking, oxidative stress, gut microbiota, gut–liver axis

## Abstract

Oxidative stress has been described as one of the main drivers of intracellular damage and metabolic disorders leading to metabolic syndrome, a major health problem worldwide. In particular, free radicals alter lipid metabolism and promote lipid accumulation in the liver, existing in the hepatic facet of metabolic syndrome, the metabolic dysfunction-associated steatotic liver disease (MASLD). Recent literature has highlighted how nicotine, especially if associated with a high-fat diet, exerts a negative effect on the induction and progression of MASLD by upregulating inflammation and increasing oxidative stress, abdominal fat lipolysis, and hepatic lipogenesis. Moreover, considerable evidence shows the central role of intestinal dysbiosis in the pathogenesis of MASLD and the impact of nicotine-induced oxidative stress on the gut microbiome. This results in an intricate network in which oxidative stress stands at the intersection point between gut microbiome, nicotine, and MASLD. The aim of this review is to delve into the molecular mechanisms linking tobacco smoking and MASLD, focusing on nicotine-induced microbiota modifications and their impact on MASLD development.

## 1. Introduction

As the most prevalent liver disorder worldwide—affecting approximately 34% of the global population—metabolic dysfunction-associated steatotic liver disease (MASLD) is responsible for a substantial proportion of liver-related health complications and has become a significant global health concern [1]. Formerly known as nonalcoholic fatty liver disease (NAFLD), the term MASLD reflects an evolving understanding of its strong link to metabolic syndrome, extending beyond the exclusion of alcohol as a contributing factor [2]. The nomenclature shift underscores the interconnection between liver steatosis and broader metabolic disturbances, such as insulin resistance, obesity, dyslipidemia, and hypertension [3], and aims to enhance disease management, improve risk stratification, and develop targeted therapies [4].

Metabolic syndrome is a cluster of conditions—including hypertension, hyperglycemia, central obesity, and abnormal lipid profiles—all increasing the risk of cardiovascular disease, stroke, and type 2 diabetes. These conditions share a common trigger: systemic inflammation, which is also central to the development of MASLD, underlying the close link between liver steatosis and metabolic syndrome [5,6,7].

Despite global efforts to reduce tobacco use, over 1.25 billion adults continue to smoke, posing ongoing public health challenges [8]. Nicotine, a primary component of tobacco, has been linked to various mechanisms that exacerbate metabolic syndrome, including disruptions in the gut microbiota and elevated oxidative stress [9,10].

Oxidative stress plays a crucial role in the progression of MASLD, acting as a key mediator of cellular damage [11]. Nicotine and other compounds in tobacco smoke (including electronic cigarettes—e-cigarettes) increase the production of reactive oxygen species (ROS) and deplete antioxidant defenses [12,13,14]. This oxidative damage amplifies the inflammatory cascade and promotes the transition from simple steatosis to more severe liver pathologies, including fibrosis and cirrhosis [15,16]. Furthermore, oxidative stress is closely linked to other hallmarks of the metabolic syndrome, making it a central factor in the onset and progression of MASLD. Recent studies have further elucidated nicotine’s harmful effects on gut health and its role in promoting oxidative imbalance. Nicotine disrupts the gut microbiome, increasing intestinal permeability and facilitating the translocation of endotoxins into circulation. Given that the gut–liver axis plays a pivotal role in metabolic health, the influence of nicotine on both gut integrity and liver function becomes particularly relevant in the context of MASLD [17].

This review seeks to explore the complex interactions between oxidative stress, gut microbiota, and nicotine within the framework of metabolic syndrome, with a specific focus on MASLD. By critically examining recent research, we aim to uncover the molecular mechanisms underpinning these relationships and assess potential therapeutic interventions. Diet, nicotine exposure, and oxidative stress are key factors influencing the gut–liver axis, and understanding their interplay is crucial for developing more effective treatments and prevention strategies for MASLD.

## 2. Oxidative Stress and MASLD

MASLD etiopathogenesis has been largely investigated for decades, and it is nowadays recognized as multifactorial. According to the historical “two-hit model” theory, first formulated in 1998 by Berson et al. [18], disease development requires two hits, the first inducing steatosis, the second represented by a source of oxidative stress able to initiate significant lipid peroxidation. The “first hit” refers to an initial metabolic disruption that raises the influx of non-esterified fatty acids (NEFA) and stimulates de novo lipogenesis. The “second hit” involves oxidative stress, reduced hepatic adenosine triphosphate (ATP) production, and the activation of pro-inflammatory cytokines, which cause necroinflammation and drive the progression to steatohepatitis [19]. Insulin resistance plays an important role in this scenario because it results in increased glucose/insulin levels and persistent hepatic lipogenesis, impairing NEFA degradation, thus representing a major pathophysiological factor in MASLD [20]. Insulin resistance is thought to be implicated in both the initiation of the disease and the progression to more advanced forms. Subsequently, the first model was revised in a “multiple hits” hypothesis, with multiple alterations acting simultaneously.

Among various factors, oxidative stress plays a key role, causing an imbalance between the liver’s antioxidant protection system from excessive levels of pro-oxidants (ROS and/or reactive nitrogen species, RNS) and free radical production in the gut and adipose tissue, resulting in hepatocyte apoptosis and tissue damage [21,22]. Significant amounts of ROS—chemical species with one unpaired electron, derived from molecular oxygen—are produced within mitochondria during molecular processes like oxidative phosphorylation, as it generates ATP, superoxide anions, and hydrogen peroxide (H_2_O_2_) as byproducts [23], and during mitochondrial β-oxidation of fatty acids when nicotinamide adenine dinucleotide (NADH) and dihydroflavine-adenine dinucleotide (FADH2) exceed the flux of oxidative phosphorylation in the electron transport chain (ETC). H_2_O_2_ is also produced during peroxisomal fatty acid β-oxidation as a physiological product. In peroxisomes, fatty acid oxidation is promoted by the acyl-CoA oxidase superfamily, which transfers electrons directly to oxygen to form H_2_O_2_ [24]. In addition, the endoplasmic reticulum can also produce ROS [25]. On the other hand, antioxidant defense systems are composed of enzymatic and nonenzymatic components. The enzymatic components comprise a collection of enzymes that effectively counteract ROS production, like α-dioxygenase, ascorbate peroxidase, superoxide dismutase, catalase, glutathione peroxidase, and glutathione (GSH) reductase [26]. Nonenzymatic elements include small molecules such as GSH, ascorbic acid (vitamin C), retinol (vitamin A), melatonin, and tocopherol (vitamin E). These molecules act as electron acceptors and protect biomolecules and cellular structures from ROS-induced damage.

Mitochondria, being a major cellular source of ROS and an organelle responsible for lipid metabolism, serve as the primary generator of reactive species in the development of MASLD [22]. Within the liver, they play a role in hepatic-specific anabolic pathways like de novo lipogenesis and gluconeogenesis. During gluconeogenesis, non-carbohydrate substrates such as lactate, glycerol, and glucogenic amino acids are converted into gluconeogenic precursors. In the mitochondria, pyruvate can be carboxylated to oxaloacetate, which is subsequently reduced to malate or transaminated to aspartate to be transported into the cytoplasm. Once in the cytoplasm, malate is reoxidized to oxaloacetate and converted to phosphoenolpyruvate by the enzyme phosphoenolpyruvate carboxykinase (PEPCK), continuing the pathway to produce glucose. When cellular energy levels are high, citrate produced in the tricarboxylic acid (TCA) cycle (derived from acetyl-CoA and oxaloacetate) can be exported from the mitochondria to the cytoplasm. In the cytoplasm, citrate is converted into acetyl-CoA and oxaloacetate by the enzyme ATP-citrate lyase [27]. Cytoplasmic acetyl-CoA is used for de novo lipogenesis, leading to the synthesis of fatty acids that can be esterified into triglycerides for energy storage [28]. Mitochondria also have a role in nonspecific one-carbon metabolism and in catabolic pathways such as TCA and urea cycles, β-oxidation, ketogenesis, and the ETC [29].

Several studies investigated the correlation between liver fat accumulation and mitochondrial impairment. For instance, Fromenty et al. described how the progressive decline of mitochondrial function, in particular β-oxidation of fatty acids, correlates with microvesicular steatosis and ultimately to hepatic disease progression [30]. Teodoro et al. studied how lipid accumulation in the liver affects mitochondrial bioenergetics in an animal model. Interestingly, after 12 weeks, oxidative phosphorylation became more efficient, indicating an early adaptive response aimed at counteracting the negative effects of triglycerides in the liver. However, by 16 weeks, mitochondrial dysfunction became clear, along with reduced activities of respiratory chain enzymes. This loss of mitochondrial efficiency was linked to the accumulation of oxidized proteins in both tissue and mitochondrial fractions. These findings suggest that fat accumulation significantly impairs mitochondrial function but also initiates a compensatory mechanism that attempts to manage and potentially reverse the bioenergetic challenges posed by steatosis [31]. Although the liver tries to compensate for fat accumulation, over time, mitochondrial adaptation becomes inadequate to stop lipotoxicity caused by the ongoing buildup of NEFA.

Such results demonstrate the existence of an interplay between molecular and biochemical disturbance in mitochondrial dynamics associated with MASLD [32,33], and oxidative stress has been hypothesized to be either a cause or a consequence of mitochondrial dysfunction accompanying MASLD development [34]. The link between oxidative stress and mitochondrial dysfunction derives from a key observation: increased mitochondrial activity in the liver has been suggested as an adaptive response mechanism to increased liver accumulation of lipids [35,36]. Indeed, high-fat diets and lipid metabolism disruption lead to hepatic accumulation of NEFA and triglycerides [37], inducing the liver to a metabolic shift to overcome the hepatic NEFA burden [38,39]. Thus, mitochondrial fatty acid oxidation and, consequently, the TCA cycle and oxidative phosphorylation are stimulated, leading to an increased flow of reducing equivalents into the mitochondrial ETC [40]. This excessive reduction in the respiratory complexes encourages the production of superoxide anion [41], which is then enzymatically transformed into H_2_O_2_ [42,43], potentially resulting in mitochondrial damage and/or triggering cellular signaling pathways. Interestingly, this pro-oxidative state seems to occur before significant mitochondrial damage in the progression of MASLD. Such alterations increase lipid peroxidation, destabilizing the inner cell redox environment [34,44]. If protracted, such an altered state could be the main contributor to hepatic mitochondrial dysfunction [35,45]. Also, a reduction in the expression and activity of ROS detoxification systems has been observed in both in vitro and in vivo studies [46]. Consequently, an excess of ROS/RNS combined with a weakened antioxidant defense emerges in MASLD.

Moreover, extramitochondrial oxidation mediated by enzymes like nicotinamide adenine dinucleotide phosphate (NADPH) oxidase, xanthine oxidase, cyclooxygenases, lipoxygenases, and inducible nitric oxide synthase (iNOS) [47] also contributes to the elevated ROS/RNS production in MASLD [48]. Notably, cardiolipin, a unique phospholipid found in the inner mitochondrial membrane, is highly vulnerable to oxidative damage. When cardiolipin is oxidized, it alters the membrane fluidity, causing destabilization and loss of ETC complex activity and the induction of mitochondrial permeability transition pore opening [49]. Additionally, the release of cytochrome C from cardiolipin into the cytosol can activate the caspase-dependent apoptotic pathway, leading to cell death [50]. Finally, oxidative stress is also linked to mitochondrial genome alterations, which are particularly vulnerable to oxidative damage because of their close proximity to ROS production sites and their lack of protective histones or efficient Deoxyribonucleic Acid (DNA) repair mechanisms. Additionally, oxidative damage to nuclear DNA can further exacerbate mitochondrial dysfunction by disrupting the transcription of essential mitochondrial proteins. This leads to a decreased expression of key regulatory factors involved in mitochondrial metabolism and biogenesis in MASLD [41,51]. These mechanisms could thereby cause a vicious cycle of mitochondrial oxidative damage and mitochondria-originating oxidative stress. Reduction in oxidative stress might be investigated as a novel therapeutic strategy to treat MASLD and its progression to steatohepatitis.

**Figure 1:**
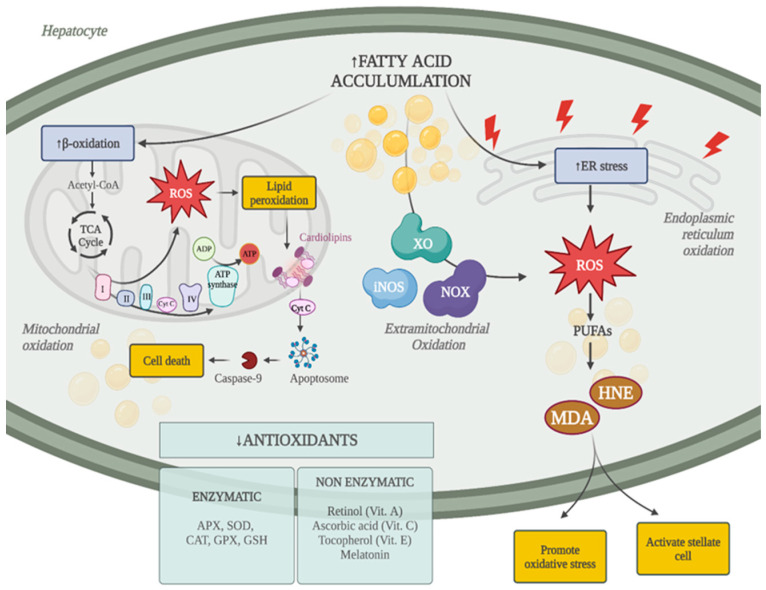
Oxidative stress and MASLD. ADP: adenosine diphosphate; APX: ascorbate peroxidase; ATP: adenosine triphosphate; CAT: catalase; CytC: cytochrome c; GPX: glutathione peroxidase; GSH: glutathione; HNE: 4-hydroxynonenal; iNOS: inducible nitric oxide synthase; MDA: malondialdehyde. NOX: NADPH oxidases; PUFAs: polyunsaturated fatty acids; ROS: reactive oxygen species; SOD: superoxide dismutase; TCA: tricarboxylic acid; XO: xanthine oxidase.

Mechanisms inducing oxidative stress in response to fatty acid accumulation in the liver are schematically represented in Figure 1.

## 3. Nicotine and MASLD

An imbalance between free radicals and antioxidant production is also the ultimate result of the pleiotropic effect that tobacco exerts on the liver, contributing to chronic liver injury. As summarized in a recent review by Premkumar et al., tobacco increases the production of pro-inflammatory molecules, such as interleukins (IL)-1 and 6 and tumor necrosis factor α, and induces the NADPH oxidase system, impairing GSH metabolism [52].

Several studies have investigated the relationship between cigarette smoking and MASLD, achieving quite heterogeneous results [53,54,55,56,57]. The first meta-analysis on the topic, including 12 observational studies and over 20.000 subjects, was published in 2018 by Akhavan Rezayat et al., showing a significant association between NAFLD and smoking (pooled Odds Ratio—OR—1.110, 95% Confidence Interval—CI—1.028–1.199). However, after performing a subgroup analysis, the association was considerable in former smokers but not in current ones, and a possible role of weight gain after smoking cessation has been hypothesized to explain such surprising findings [58]. More recently, another meta-analysis by Zhang et al. confirmed that smoking cessation was not a protective factor against NAFLD, whose prevalence did not decrease in former compared to current smokers [59]. Results about the progression to advanced disease stages, instead, are more consistent, with researchers agreeing that cigarette smoking is associated with steatohepatitis and hepatic fibrosis. In particular, a large study on 1091 subjects with biopsy-proven nonalcoholic steatohepatitis first showed that a higher cigarette consumption (≥10 pack-years) was more common in patients with advanced fibrosis [60]. Such a dose-dependent relationship was later confirmed in other cohorts of patients [58,61].

Interestingly, not only active but also passive smoking proved to be associated with hepatic steatosis. In the aforementioned meta-analysis by Akhavan Rezayat et al., passive smoking was associated with NAFLD with an OR of 1.380 (95% CI, 1.199–1.588) [58]. Cotinine, nicotine’s main metabolite, is a widely recognized biomarker of smoking status thanks to its long half-life, typically ranging between 8 to 30 h, which makes it largely more reliable than nicotine itself, having a mean half-life of only 2 h. It allows the distinction between non-smokers and passive and active smokers, and specific cut-offs have been identified for different ethnicities and particular conditions (e.g., pregnancy or disease states) [62]. In a recent population-based study including 1433 teenagers from the United States, She et al. used cotinine serum levels to assess the smoking status and found a positive association between both active and passive smoking and Liver Stiffness Measurement—estimating the degree of fibrosis—but surprisingly, no association was observed with Controlled Attenuation Parameter, an ultrasonographic parameter for steatosis assessment [63]. Another study from the United States on a large cohort of more than 7400 adult non-smokers exposed to passive smoking has demonstrated a linear dose-response relationship between cotinine levels and the risk of NAFLD, stressing the importance of measures to reduce secondhand smoke exposure [64]. Furthermore, growing evidence shows that smoke toxins accumulated in different materials or deposited on surfaces—the so-called “thirdhand smoke”—may also have a harmful impact on human health, including NAFLD development [65,66].

It has been observed that smoking not only induces liver damage per se but also acts as a booster for high-fat diet-related liver injury, further highlighting the crucial importance of a healthy lifestyle. Azzalini et al. analyzed the impact of cigarettes on a genetic model of obese rats vs. control ones: after smoke exposure, the obese group showed increased alanine transaminase serum levels and a worsening in histological features typical of NAFLD (hepatocellular ballooning and lobular inflammation), whereas milder changes were detected in control rats. Moreover, they demonstrated in obese smoker rats an increase in two markers of oxidative stress, the hydroxynonenal and carbonylated proteins [67]. Similar results showing that obese rats were more susceptible to smoking-induced liver damage were confirmed by other research groups [68,69]. A combined action of nicotine and a high-fat diet seems to be the key player in oxidative stress, together with hepatocellular apoptosis, impaired endoplasmic reticulum function, and disrupted lipid homeostasis, leading to hepatic lipogenesis [70].

## 4. Gut Dysbiosis and MASLD

Intestinal dysbiosis is implicated in various liver diseases, including MASLD [71,72], defining the so-called “gut–liver axis”—a term referring to the close communication between the intestine and liver, ensuring a defense system against pathogens [73]. In healthy conditions, the intestinal barrier made up of mucus from goblet cells and tightly connected epithelial cells (through claudins and occludins) works together with the local immune system as a defense against pathogens. Dysbiosis causes intestinal wall damage, leading to increased permeability and promoting systemic inflammation [74,75]. In this endotoxemic state, driven by the activation of the lipopolysaccharide (LPS)/Toll-like receptor 4 (TLR4)/nuclear factor-κB (NF-κB) signaling pathway, Kupffer cells (liver macrophages) and hepatic stellate cells are also activated. This triggers an inflammatory response that spreads to the liver, leading to hepatic fibrosis and impairing the production of short-chain fatty acids (SCFAs). These SCFAs are essential for preserving the intestinal barrier and regulating immune functions. As a result, the weakened intestinal barrier allows more bacterial components, such as LPS, to enter the bloodstream, further exacerbating inflammation and liver damage in a self-perpetuating cycle [76,77,78,79]. These mechanisms indeed explain why MASLD appears more common in conditions typically characterized by the disruption of the intestinal barrier, notably inflammatory bowel diseases. In such conditions, as we described in a recent review paper, a reduced amount of SCFAs is associated with increased levels of pro-inflammatory metabolites like cytokines and bacteria-derived LPS, whose translocation from the bowel to the liver interferes with hepatic lipid metabolism, promoting fat accumulation [80].

Current evidence demonstrates that MASLD patients show different microbiota profiles compared to healthy subjects. More specifically, at a phylum level, an increased abundance of Gram-negative Bacteroidetes has been observed in MASLD patients, resulting in a reduced Firmicutes/Bacteroidetes (F/B) ratio, whose balance is regarded as a marker of intestinal homeostasis [17,81]. The rise in Bacteroidetes leads to increased methanol production, which can accumulate and cause oxidative damage in the gut and liver [82,83], a process that may be further worsened by alcohol-producing strains of *K. pneumoniae* [84,85]. MASLD patients also show increased levels of Proteobacteria. In a cross-sectional study on 37 patients with MASLD, Jasirwan et al. found a direct link between the F/B ratio and steatosis, while Proteobacteria strongly correlated with fibrosis [86]. The rise in Proteobacteria is negatively correlated with blood histidine levels, which play an important role in cellular protection against oxidative stress [72,87]. Indeed, only histidine supplementation has been demonstrated to improve MASLD in murine models [88].

It has also been observed that patients with MASLD show an increase in *Enterobacterales*, linked to higher production of trimethylamine [72,89,90,91]. Trimethylamine is transported via the enterohepatic circulation to the liver, where it is metabolized into trimethylamine N-oxide (TMAO), a potential risk factor for MASLD and hepatic fibrosis [92,93]. Recently, Hai et al. investigated the role of microbiota-derived TMAO in MASLD patients. They found that TMAO levels correlated with increased serum levels of IL-33 and its receptor suppression of tumorigenicity 2, both in human samples and mouse models, while IL-33 deficiency was associated with a reduced abundance of TMAO-producing bacteria. Interestingly, the inhibition of TMAO synthesis mitigated oxidative stress, and fecal transplantation from IL-33 deficient donors prevented MASLD progression in wild-type mice [94].

Numerous studies have also demonstrated a reduction in *Faecalibacterium prausnitzii* in patients with MASLD [95,96]. This bacterium is the most important butyrate producer in the intestine and is considered an indicator of human health [97]. Studies on murine models have shown that treatment with *F. prausnitzii* reduces hepatic fat content and fibrosis [96,98,99]. Additionally, a reduction in *Akkermansia muciniphila* has been reported, and its supplementation has been correlated with reduced hepatic steatosis by enhancing the oxidation of accumulated lipids [72].

**Figure 2:**
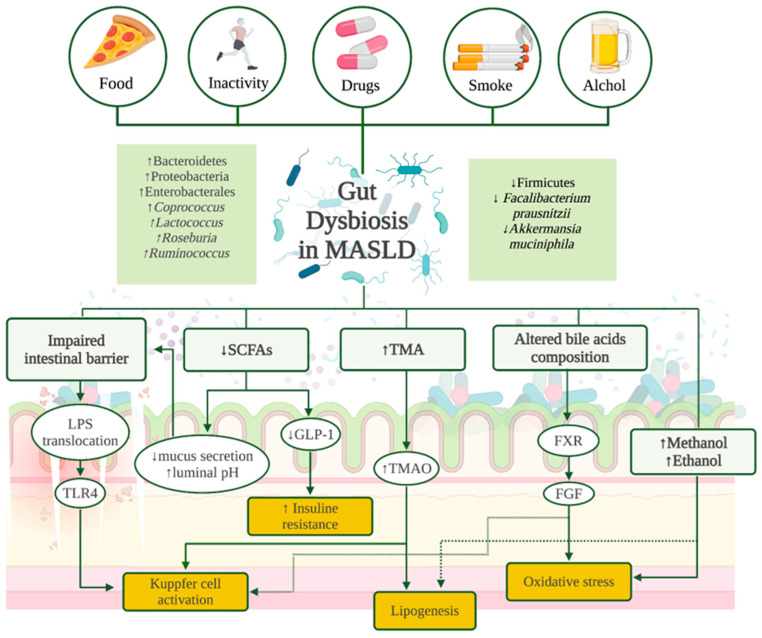
Gut dysbiosis in MASLD. FGF: fibroblast growth factor; FXR: farnesoid X receptor; GLP-1: glucagon-like peptide-1; LPS: lipopolysaccharide; MASLD: metabolic dysfunction-associated steatotic liver disease; SCFAs: short-chain fatty acids; TLR4: toll-like receptor 4; TMA: trimethylamine; TMAO: trimethylamine N-oxide.

Alterations in the gut microbiota can disrupt the production of SCFAs such as acetate, propionate, and butyrate, which possess anti-inflammatory properties, enhance mucus secretion, lower luminal pH, and supply energy to enterocytes. Additionally, they stimulate the production of gut hormones like glucagon-like peptide-1, which plays a role in regulating blood glucose levels, insulin secretion, and inflammation [100,101]. Indeed, studies in murine models have demonstrated that SCFA supplementation can alleviate steatosis and inflammation [102,103]. Additionally, dysbiosis influences bile acid metabolism, affecting farnesoid X receptor (FXR) signaling, which is involved in regulating lipid and glucose metabolism in the liver [104]. Under healthy conditions, hepatocytes generate bile acids that are conjugated and then transported to the ileum, where they are metabolized by the gut microbiota, leading to the production of secondary bile acids [105]. Different studies showed that a high-fat diet associated with MASLD alters the profile of primary bile acids [106,107,108]. This shift impacts the gut microbiota, leading to higher levels of *Coprococcus*, *Lactococcus*, *Roseburia*, and *Ruminococcus* [109]. FXR is highly expressed in the liver and intestine, where it regulates bile acid enterohepatic circulation and interacts with fat metabolism [110,111]. It has been demonstrated that intestinal microbiota can alter bile acid secretion via FXR, leading to increased lipid peroxidation and hepatic steatosis [104,112,113]. Figure 2 summarizes the main microbiota shifts in MASLD and their molecular effects.

Therefore, the intricate relationship between dysbiosis and MASLD underscores the vital importance of the gut–liver axis in maintaining metabolic health. The growing understanding of MASLD-specific microbiota signatures and their impact on metabolic pathways is of utmost importance to define new potential therapeutic strategies. Several approaches have been suggested to reestablish a balanced gut microbiota and improve MASLD outcomes. Adjustments in lifestyle, especially dietary habits, indicate that a diet with a high fiber content not only lowers cholesterol, blood sugar levels, and insulin resistance but also boosts *Akkermansia* levels, resulting in increased production of SCFAs, which are crucial for preserving the integrity of the intestinal barrier [114,115,116,117,118]. Moreover, FXR has emerged as a promising therapeutic target due to its critical role in regulating bile acid metabolism, maintaining gut–liver homeostasis, and modulating inflammatory and fibrotic processes. Targeting FXR could help break the vicious cycle of inflammation, intestinal barrier dysfunction, and liver damage, offering potential benefits in managing endotoxemia-related conditions [119,120]. Gut microbiota modulation through probiotics is also a practical strategy for tackling the imbalance in the gut–liver axis. Importantly, multiple randomized clinical trials have indicated that fecal microbiota transplantation (FMT) may be an effective treatment for MASLD and obesity [121,122,123]. A recent study by Liu et al. found that apolipoprotein H is vital for regulating metabolism and gut microbiota. Lower levels of this protein may lead to hepatic steatosis by boosting fat production and causing intestinal dysbiosis with reduced microbiota diversity [124]. These findings highlight apolipoprotein H as a possible therapeutic target and emphasize the importance of combining lifestyle changes with drug treatments to restore the gut–liver axis. Microbiota-related potential therapeutic targets are schematically summarized in Table 1.

## 5. Effects of Cigarette and E-Cigarette Smoking on Gut Microbiota

Disruptions of the gut barrier may derive from different lifestyle factors, such as physical inactivity, unhealthy diet, smoking, and alcohol consumption [10]. These changes heighten systemic inflammation, ultimately leading to intestinal dysbiosis.

The influence of cigarette smoking on gut microbiota is a major focus of scientific research and remains not fully understood. While it is well-established that smoking induces oxidative stress on the intestinal wall [125], recent observations suggest that smoking might also alleviate ulcerative colitis symptoms in mouse models by positively influencing the composition of the intestinal flora [126].

There is a strong association between cigarette smoking and the decrease in the biodiversity of gut microbiota, primarily promoting the growth of Proteobacteria, Bacteroidetes, *Clostridium*, and *Prevotella* while significantly reducing the presence of *Actinobacteria* and Firmicutes [127]. A reduced F/B ratio is observed, as previously described in the context of MASLD. More specifically, a study conducted by Huang et al. demonstrated that exposure to cigarette smoke leads to an upregulation of TLR-4, NF-κB, and the myeloid differentiation primary response 88 (MyD88) signaling pathway, triggering a harmful inflammatory response in the intestinal wall. This inflammation results in a reduction in Firmicutes, *Lactobacillus*, and *Akkermansia*, along with an increase in *Helicobacter* and Bacteroidetes. Notably, the study identified the NF-κB signaling pathway as a potential breakthrough target in the treatment of smoke exposure-mediated lung and gut injury [128]. Consistently, an increase in Firmicutes and *Actinobacteria*, as well as a reduction in Bacteroidetes and Proteobacteria, has been observed after quitting smoking [129,130,131].

Interestingly, Whitehead et al. demonstrated that cigarette smoking causes sex-dependent effects, notably distinct changes in gut microbiota between female and male mouse models. Indeed, they found a decrease in α-diversity in female mice compared to male ones. Moreover, the F/B ratio was lower in male mice, while in female mice, it remained closer to the physiological state [132]. This may explain why some human studies have not shown a clear increase in Bacteroidetes, possibly due to not considering gender differences [131].

The gut microbiota also plays a significant role in influencing nicotine addiction. A study by Chen Y. et al. highlighted that *F. prausnitzii* offers protection against nicotine dependence. In contrast, *Eubacterium xylanophilum*, *Lachnoclostridium*, and *Holdemania* appear to promote smoke addiction in human beings [133].

Such results refer to traditional tobacco smokers but cannot be extended to e-cigarette users. Indeed, the latter does not show an increase in *Prevotella*, and their microbiota appears similar to non-smokers’ [131,134,135]. With the spread of e-cigarettes and other forms of cigarettes, a deeper understanding of their potential intestinal effects becomes crucial, particularly in investigating whether there are or are not substantial differences in comparison to non-smokers.

## 6. The Intersection Point: Cigarette Smoking, Oxidative Stress and Gut–Liver Axis

The liver’s particular location, at the junction between the portal blood flow from the intestinal circulation and other peripheral organs, leads to a close interaction with the gut, justifying the expression “gut–liver axis” [136]. While the gut supports liver health and metabolism with microbes and their metabolites, nutrients, and gut-derived hormones, the liver influences gut homeostasis by producing bile acids, which eventually help regulate the gut–liver axis, thus establishing a bidirectional communication [137,138]. A healthy commensal microbiota plays a protective role for the liver and helps prevent hepatic damage. Conversely, in the case of dysbiosis, specific gut bacteria such as *Escherichia coli* and *Enterococcus faecalis*, along with microbial metabolites like LPS from Gram-negative bacteria, peptidoglycans from bacterial cell walls, and flagellin from bacterial flagella, interact with the gut epithelium. These molecules can cross the compromised intestinal barrier and translocate to the liver via the portal vein system. For instance, LPS, a component of Gram-negative bacteria like *Escherichia coli*, binds to TLR4 on Kupffer cells, triggering inflammation and oxidative stress. Peptidoglycans from *Enterococcus faecalis* can activate innate immune responses, contributing to liver damage. Flagellin, derived from motile bacteria, activates TLR5 signaling, exacerbating liver inflammation. This cascade generates ROS, amplifying oxidative stress and driving inflammatory processes that result in liver injury, fibrosis, and the progression of liver diseases like NAFLD or alcoholic liver disease [139]. Mazagova et al. explored whether the lack of intestinal microbiota leads to variations in liver inflammation by analyzing germ-free (GF) and Myd88/Trif-deficient mice (lacking downstream TLR signaling) [140]. In GF mice, due to the lack of bacteria, they observed increased liver fibrosis and a severe phenotype, including altered metabolic reactions, vitamin deficiencies, and dysregulation of the immune system. In Myd88/Trif-deficient mice, hepatocytes were identified to be more susceptible to toxin-induced cell death. To better understand the mechanism behind the increased liver fibrosis in GF mice, researchers measured plasma levels of indole-3-propionic acid (IPA), a product of dietary tryptophan with a powerful antioxidant activity, whose production is completely dependent on the presence of *Clostridium sporogenes* in gut microflora [141]. They discovered that GF mice had no detectable IPA in their plasma compared to conventional mice. The lack of IPA may make hepatocytes more vulnerable to cell death, confirming the protective role of commensal microbiota in preventing fibrosis in chronic liver injury. Mazagova et al. showed that a low baseline level of bacterial products may be adequate to protect the liver from toxic stimuli. In fact, microbial-derived IPA may offer hepatic protection against oxidative stress, while elevated systemic levels of microbial products are unlikely to offer additional protection. Instead, they may activate hepatic stellate cells and Kupffer cells or recruit macrophages, leading to increased liver damage. Their findings highlight an often-overlooked protective aspect of the gut–liver axis. This microbiota-mediated protection enhances hepatocyte resistance to damage and bolsters their self-defense mechanisms [140].

Due to such a beneficial antioxidant action of some commensal bacteria, researchers’ interest has turned to the potential relationship between gut microbiota disorders and liver function injury induced by cigarette smoking. Currently, most data come from pre-clinical studies on mouse models. In their interesting work, Meng et al. examined the influence of cigarette smoking on gut microbiota and liver in mice exposed to cigarette smoking compared to mice inhaling normal air (control group). Consistent with previous data reporting the effect of nicotine on body weight loss in both humans and animals [142,143,144,145], this study confirmed how cigarette smoke exposure decreases body weight by reducing appetite and food intake and inducing a significant reduction in serum lipid levels, such as serum total cholesterol, high-density lipoprotein cholesterol (HDL-C), and low-density lipoprotein cholesterol levels. Simultaneously, smoke-exposed mice showed an altered bacteria composition associated with an altered liver transcriptome, notably an impaired expression of lipid metabolism-related genes. The subsequent disrupted lipid metabolism and liver injury were demonstrated by increased serum total bilirubin in the smoke-exposed group, as well as higher serum transaminase levels, indicating that cigarette smoke exposure eventually induces liver damage [146].

Therefore, preserving a balanced microbiota may represent a potential strategy to mitigate oxidative stress-related liver injuries, and microbiota modulation has been proposed as a complementary approach to treating chronic liver disease [147,148]. Emerging evidence has underlined the antioxidant action of some probiotics, such as *Lactobacillus johnsonii* and *Lactobacillus plantarum*, able to reverse changes in hepatic oxidative pathways and restore microbiota balance [149,150]. The potential role of *Bacillus* species as probiotics has also gained a growing scientific interest in recent years. After observing the beneficial effects of *Bacillus amyloliquefaciens* SC06 and *Bacillus licheniformis* SC08 in alleviating oxidative stress-induced intestinal disorders and apoptosis [151], Wu et al. further investigated whether the oral administration of these two *Bacillus* species may prevent liver injuries in rats injected with diquat [1,10-ethylene-2,20-dipyridylium], a potent pro-oxidant widely used both in vivo and in vitro studies to induce oxidative stress [152]. *Bacillus* SC06 as a probiotic demonstrated to be useful to alleviate both oxidative stress-induced liver and intestinal injury, showing a greater antioxidant activity compared to SC08, as indicated by the improved liver function, reduced mitochondrial dysfunction, and increased antioxidant levels following diquat exposure, along with the capacity to modulate the defensive system upon oxidative stress. Interestingly, smoke-induced microbiota changes may be specifically addressed to alleviate liver injury in smokers or smoke-exposed subjects. In their study, Meng et al. described an increase in the relative abundance of *Eubacterium* and *Lactobacillaceae* and a decrease in the relative abundance of *Lachnospiraceae*, especially of *Salmonella,* in smoke-exposed mice [146]. Liu et al. examined the difference in cecal microbiota between two Japanese quail strains fed with either a control diet or a cholesterol-enriched diet to determine how cecal microbiota can be modified by host genotype and diet and if it may play a part in cholesterol metabolism. The two strains were, respectively, atherosclerosis-susceptible and -resistant; after 6 weeks on their respective diets, the cecal microbiota of 12-week-old quail was analyzed, evidencing that in resistant quail strains on a control diet, there was a positive correlation between the abundance of *Lachnospiraceae* and plasma HDL-C levels [153]. Another study by Sun et al. conducted in diet-induced hypercholesterolemic hamsters fed with oat-based food showed that *Eubacterium bacteria* could metabolize cholesterol [154]. Thus, oral administration of the probiotic *Lactobacillus* or its mixture demonstrated in mice the capacity to lower cholesterol levels, improving liver steatosis and dyslipidemia caused by a high-fat diet [155]. These findings suggest that changes in gut microbiota could play a role in regulating lipid metabolism after cigarette smoke exposure.

## 7. Conclusions

The presented scientific evidence underscores the crucial role of the gut–liver axis and gut microbiota in the pathogenesis and progression of MASLD. Risk factors such as cigarette smoking, poor diet, physical inactivity, and exposure to toxic agents disrupt gut microbiota balance, increasing oxidative stress, dysbiosis, and systemic inflammation. Cigarette smoking significantly modulates the gut microbiota by reducing bacterial diversity and promoting pro-inflammatory species like Bacteroidetes and Proteobacteria. These changes, combined with the activation of the TLR4/NF-κB pathway, exacerbate inflammation, impair the intestinal barrier, and facilitate microbial translocation and hepatic lipid accumulation. Interestingly, certain studies suggest the protective effects of smoking in specific conditions, such as ulcerative colitis, highlighting a complex biological interplay that remains unclear. MASLD patients exhibit specific microbiota profiles, including reduced levels of beneficial bacteria such as Faecalibacterium prausnitzii and Akkermansia muciniphila and an increase in species like Enterobacterales and TMAO-producing bacteria. These changes amplify oxidative stress, hepatic inflammation, and metabolic disturbances, aggravating steatosis and fibrosis. Therapies targeting gut microbiota, such as probiotics, prebiotics, and fecal microbiota transplantation (FMT), are gaining traction as complementary MASLD treatments. Additionally, molecular targets like the FXR receptor and key proteins such as apolipoprotein H offer promising therapeutic potential. Further research should focus on understanding the interplay between environmental factors (e.g., smoking, diet), genetics, and microbiota in MASLD pathogenesis. Precision medicine represents a critical frontier, enabling personalized interventions based on microbiota profiles and molecular characteristics, combining probiotics, FXR-targeting drugs, and lifestyle modifications. The growing use of e-cigarettes and heated tobacco products requires investigation to determine their impact on gut microbiota and liver health compared to traditional cigarettes.

Elucidating the mechanisms underlying intestinal dysbiosis, oxidative stress, and metabolic dysfunction in the gut–liver axis could transform MASLD management. By integrating preventive strategies (healthy lifestyle) with innovative therapies (microbiota-targeting and metabolic drugs), clinical outcomes for MASLD patients could be significantly improved.

## Figures and Tables

**Table 1:** Microbiota-related potential therapeutic approaches for MASLD.

Reference	Type of Study	Population	Approach	Benefits
Evans C.C., 2014 [114]	Prospective	Mouse model	Exercise	Inverse correlation between run distance and Bacteroidetes ratio (r^2^ = 0.35, *p* = 0.043)
Carbajo-Pescador S, 2019 [116]	Prospective	Mouse model	Exercise	-Reduction in HFD-induced hepatic steatosis by modulating lipid metabolism -Increase in beneficial bacteria (Parabacteroides, Bacteroides, *Flavobacterium*) and decreased harmful ones (*Blautia, Dysgonomonas*, *Porphyromonas*).
Campbell SC, 2016 [117]	Prospective	Mouse model	Diet and exercise	Increase in *Faecalibacterium prausnitzii*, *Clostridium* spp., and *Allobaculum*
Ding L, 2021 [120]	Prospective	Mouse model	Ft1 (TGR5 agonist)	Ft1 activates TGR5 and inhibits FXR, alleviating high-fat diet-induced obesity and insulin resistance in mice.
Zoll J, 2020 [121]	Prospective	Mouse model	Effects of FMT on exercise	FMT from any donor group did not alter body composition in recipient mice.
Pérez-Matute P, 2020 [122]	Prospective	Mouse model	FMT	FMT potentiates weight loss and increases bacterial richness and diversity.
Lai ZL, 2018 [123]	Prospective	Mouse model	FMT	-FMT increases *Helicobacter*, *Odoribacter,* and AF12 -FMT improves metabolic profiles
Liu Y, 2023 [124]	Prospective	Mouse model	APOH	APOH downregulation causes lipogenesis, hepatic steatosis, and dysbiosis

HFD: high-fat diet; FMT: fecal microbiota transplantation; APOH: apolipoprotein H; Ft1: notoginsenoside Ft1; TGR5: membrane-bound G protein-coupled receptor; FXR: farnesoid X receptor.

## Data Availability

No new data were created or analyzed in this study. Data sharing is not applicable to this article.
